# Early Postoperative Cardiac Complications Following Heart Transplantation -

**DOI:** 10.31661/gmj.v12i.2701

**Published:** 2023-09-12

**Authors:** Sohrab Negargar, Sahar Sadeghi

**Affiliations:** ^1^ Cardiovascular Research Center of Tabriz University of Medical Sciences, Tabriz, Iran

**Keywords:** Heart Failure, Heart Transplantation, Postoperative Complication

## Abstract

Cardiovascular disorders remain the leading cause of death around the world. Heart transplantation is considered the only therapeutic choice defined as the gold standard strategy to manage end-stage heart failure. Nevertheless, the remaining postoperative complications compromise both the survival rate and quality of life in heart transplantation recipients. The present study aimed to review the current findings concerning the main early complications after heart transplantation, reliable predictors, diagnostic approaches, novel surgical techniques, and management strategies. The results demonstrated that significant advances in immunosuppressive pharmaceuticals, determining appropriate policies for donor acceptance, pre and post-operative treatment/care, selection of the most compatible donor with the recipient, and the suggestion of novel diagnostic and surgical techniques over the past decade had dropped the mortality and morbidity rates early after transplantation. However,marrhythmia, atrial flutter, atrial fibrillation, deep sternal wound infection along with other sites infections, low cardiac output syndrome, acute graft dysfunction, pericardial effusion, constrictive pericarditis, and acute cellular rejection could be considered as the major early complications following heart transplantations that pivotally require further investigations.

## Introduction

Cardiovascular diseases (CVDs), one of the most drastic health problems and the most prevalent non-communicable diseases, are recognized as the leading cause of morbidity and mortality since they lead to more than 30% of all deaths all around the world [1][2][3].

More importantly, it is documented the mortality rate caused by CVDs continues to increase; as regards 2012 statistics, the death rate due to CVDs was 17.5 million and surged to approximately 18 million in 2016, which is expected to reach more than 22 million deaths by 2030 [4][5][6]. Indeed, mortality is claimed to be one of the most accurate incontestable CVDs outcomes as it prepares a beneficial measure of CVDs burden [7].

Along with the mentioned facts about high rates of mortality related to CVDs, this health issue is described as one of the major factors that deteriorate the quality of life at the micro-level in patients/their families as well as at the macro-level CVDs cause a critical financial burden on the health budget of countries, specifically the countries with low- and middle-incomes [7][8].

Notably, Continuous studies over recent decades have identified two major changes, including social changes and lifestyle changes, in human societies that have contributed to exponential development in CVDs incidence and progression. Social changes include prolonged office work times, physical demands and more sedentary jobs, and decreased leisure and recreation time for frivolous accomplishments, whilst lifestyle changes could be exemplified by high fat and calories rich diets, reduced physical activities, alcoholism, and smoking [9][10].

In addition, two main classifications are considered risk factors related to CVDs, which include modifiable risks (e.g., physical inactivity, metabolic syndrome, hyperglycemia, undesirable lipid profile, obesity, prolonged stressful state, hypertension, alcoholism, and smoking) and non-modifiable risks (e.g., gender, age, and familial history) [11][12].

It is documented that CVDs refer to all disorders related to the cardiac tissue and blood vessels, including hypertension, cerebrovascular disease, peripheral vascular disease, deep vein thrombosis, atherosclerosis, pulmonary embolism, cardiac arrhythmias, myocardial fibrosis, atrial fibrillation, and heart diseases such as hypertrophic cardiomyopathy, coronary heart disease, pericarditis, rheumatic heart disease, dilated cardiomyopathy, congenital heart disease, and diabetic cardiomyopathy all of which could result in heart failure [4][7].

Heart failure is described as a heterogeneous syndrome with a challenging case ascertainment [13]. The incidence of heart failure is rising worldwide due to the aging of the population. In fact, heart failure is documented as the most common reason for hospital admission of elderly people (65 years old or more) [14]. In addition, it has been prospected that approximately eight million or more individuals over 18 years old will be affected by heart failure, with a current prevalence of almost forty million patients [15].

Despite the spectacular advances during the recent three decades in CVDs medicine and surgery, which is resulted in significant patient prolonged lives, in many cases, the myocardial damage remains a lifetime, and heart disease will not cure [14].

Current therapeutic approaches and clinical cares include three evidence-based device strategies, seven evidence-based medications, and several recommend processes of care [16]. In addition to the extant imperfections, pharmaceutical approaches depend on immutable factors such as race and gender that leads to different responses to therapies in patients [17].

Along with that, heart failure is divided into three subgroups, including heart failure with reduced ejection fraction, heart failure with preserved ejection fraction, as well as heart failure with improved ejection fraction, each of which response differently to treatment methods. For example, β-adrenoreceptor blockers, renin-angiotensin-aldosterone system blockers, and angiotensin receptor-neprilysin inhibitors are documented therapies considered for heart failure with reduced ejection fraction; however, other forms of heart failure require further investigations [14][16].

Therefore, in end-stage heart failure, heart transplant surgery is considered the only therapeutic choice, which is followed by early and late postoperative consequences.

Due to the importance of this issue in both the patient’s survival rate and the patient’s quality of life, the present study has aimed to briefly introduce the cardiac transplantation procedure and then review the early postoperative consequences after heart transplantation.

## Heart Transplantation, the Final and Only Choice in End-Stage Heart Failure

In 1967, the first heart transplantation was performed by Christian Barnard in South Africa [18]. Although at first there was a significant euphoria, the outcomes of this novel method were not satisfactory due to the remarkably high rates of mortality [19].

Subsequently, at the end of the 70s, pharmaceutical agents such as cyclosporine were accompanied by this novel method which assisted remarkably in the control of postoperative conditions resulting in lower cases of rejection and the development of all performed surgeries, particularly heart transplantation [19][20]. Consequently, heart transplantation represented an impressive clinical therapeutic outcome for heart failure. All of these findings, in turn, lead to the consideration of heart transplantation as the definitive gold standard surgical approach for treating heart failure [21].

It is documented that heart transplantation is capable of improving both qualities of life and survival in patients with end-stage heart failure. Hence, despite medications and surgical procedures, heart transplantation should be considered the ideal choice for patients who maintain functional classes III and IV, exert poor prognosis markers, and have recurrent hospitalizations [22][23].

Despite all these preponderances, successful heart transplantation critically requires pre-operative considerations because there are several contraindications, such as pulmonary hypertension, severe cerebrovascular diseases, severe peripheral vascular diseases, severe liver failure, severe pulmonary disease, and ABO incompatibility in prospective donor and recipient crossmatching that lead to post-transplant consequences in the recipients [24][25].

However, even primary successful considered transplantation could be followed by subsequent complications, which are divided into early and late subgroups based on the time of occurrence [19]. In the following, the present study will review the findings regarding the early postoperative complications after heart transplantation.

## Early Complications

It is believed that post-operative complications after heart transplantation, and particularly the risk of mortality, are complex and dynamic [19].

Despite recent improvements in donor procurement, surgical techniques, immunosuppressant, and post-transplantation care, both early and long-term complications continue to threaten patients’ survival or target their quality of life [26].

In the following, the current study reviews the early postoperative complications after transplantation, describes the etiology of these destructive events, as well as mentions the current strategies to confront these complexities.

*Arrhythmia*

The increased risk of postoperative bradycardia is considered an impartible and irrefutable event in heart transplant recipients which represents the desired prognosis with pacemaker therapy [27]. Moreover, atrial tachyarrhythmias could be considered another risk for patients who underwent heart transplantation [28][29]. Early studies were limited to arrhythmias caused by pre-existing donor heart substrates like dual atrioventricular nodal physiology or atrioventricular accessory pathways [30]. However, during the last decade, performed investigations in the USA transplant centers revealed that supraventricular tachycardia could affect approximately 10% or more of cardiac transplant recipients. Moreover, they demonstrated that during heart transplant surgery, complicated modifications of the atrial substrate could be followed by both proarrhythmic and antiarrhythmic consequences which are mainly dependent on the performed surgical procedure [31][32].

Indeed, during heart transplantation, surgical modification of the atria causes antiarrhythmic consequences such as vagal denervation and pulmonary vein isolation, as well as proarrhythmic consequences, for instance, the creation of atriotomy scar [33]. In addition, pre-existing accessory pathways and/or dual atrioventricular nodal physiology in donated hearts could develop supraventricular tachycardia, which is not curable with adenosine treatment, but catheter ablation is highly recommended [33]. The most prevalent macroreentrant atrial arrhythmia is assumed to be cavotricuspid isthmus-dependent right atrial flutter which probably is caused by the isolation of the right atrial posterior wall in transplant recipients [33]. Similarly, counterclockwise cavotricuspid-dependent atrial flutter followed by atrial tachycardia is considered the most frequent late supraventricular arrhythmias in orthotopic cardiac transplantation recipients, possibly originating in border or low-voltage zones adjacent to the atrio-atrial anastomosis which are completely confrontable with radiofrequency ablation [34]. Importantly, cardiac arrhythmia could be considered a potential marker of early progression of cardiac allograft vasculopathy as well as an indication for implantation of heart pacemakers. Noworolski *et al*. retrospectively evaluated the records of more than 500 consecutive patients who underwent cardiac transplantation and found that atrioventricular block, sinus node dysfunction, and cardiac arrhythmias are possible predictors of the requirement for peacemaker implantation in heart transplantation recipients [35]. However, a permanent pacemaker implant may involve complications such as infection, metal poisoning, etc., which can be countered with recent advances such as the use of lead-free pacemakers and disinfection approaches [35][36].

In addition, both atrial fibrillation and atrial flutter are considered the most frequent atrial arrhythmias in heart transplantation recipients [37][38].

Rodríguez-Entem *et al*. revealed that in patients with symptomatic atrial flutter after orthotopic heart transplantation counterclockwise circuit around the tricuspid annulus, which involves the cavotricuspid isthmus, is the most frequent mechanism causing atrial flutter. Moreover, they suggested that catheter ablation of the isthmus between the posterior atrial suture line and tricuspid annulus could be assumed as an effective therapeutic approach [39][40]. Furthermore, an alteration in the surgical technique to a bicaval anastomosis was suggested as a preventive method [39].

Similarly, implantable cardioverter defibrillator therapies such as beta-blockers or amiodarone administration may represent beneficial consequences on the survival of heart transplantation candidates [41]. Along with that, several contemporary surgical treatments for atrial fibrillation are considered to involve creating a subset of the lesions made in the known Cox-Maze procedure, which consists of pulmonary vein isolation and partial cardiac denervation, which is common in heart transplantation recipients [42][43]. Importantly, by evaluating 498 consecutive cases, Cohn *et al*. revealed that approximately 6% of patients experienced postoperative atrial fibrillation within 60 days of transplant. Interestingly, they revealed that pulmonary vein isolation and partial cardiac denervation might protect heart transplantation recipients from postoperative atrial fibrillation [44].

## Deep Sternal Wound Infection and Other Infections

Deep sternal wound infection is considered a drastic complication after any cardiac surgery, with a prevalence of 1-10% and a significant mortality rate of 10-20% and even near 50% [45][46]. In this regard, deep sternal wound infection after heart transplantation is believed to be a life-threatening complication involving 2.5-3.6% [47]. Furthermore, a 5-year survival averaging 70-90% is documented for patients with this type of infection [48]. Due to the importance of this complexity, a group of researchers has tried to determine the risk index, which in turn enables physicians to quickly classify patients with sternal wound infection into four risk groups [49]. More importantly, in addition to the possible deteriorative effects of common immunosuppression cardiac transplant recipients, the ideal therapeutic approach remains a matter of controversy [50][51].

Along with admitting the high rates of mortality and morbidity caused by deep sternal wound infection after heart transplantation, Filsoufi *et al*. considered the risk factors to be related to prior ventricular assist device implantation and pre-operative inotropic support [52]. Through a retrospective study on 437 consecutive patients, Wallen*et al*. demonstrated that this type of infection imparts a remarkable burden on heart transplant recipients, in which virulent gram-negative bacteria were believed to be the predominant causative organisms [53]. Fortunately, in recent years, researchers have suggested several therapeutic approaches in order to tackle sternal wound infections post-heart transplantation. O’Keeffe *et al*., for example, demonstrated that the division of the sternocostal origin, as well as the humeral insertion of the pectoralis major muscle, could exert a beneficial method to increase sternal coverage [54]. In addition, vacuum-assisted closure therapy represented a suitable response in terms of granulation tissue in growth and infection decline in all of the studied patients [55]. Omental flap transposition is another therapeutic approach that Carrier *et al*. suggested as the most effective method for the treatment of recurrent deep sternal wound infection as well as mediastinitis after any cardiac surgery with acceptable mortality, low morbidity, and appropriate late results [56]. In recent years, the dehiscence of sternal wound infection via extracellular matrix peach and effective diagnosis by metagenomic next-generation sequencing are assumed as other types of tackling this common early complication after heart transplantation [57][58]. Furthermore, Lin *et al*. revealed that unilateral pedicled pectoralis major transfer, harvested by endoscopic-assisted method, provides a simple, reliable, and straightforward procedure to manage both sternal infection and mediastinal obliteration without any violation of the second flap [59].

In addition to sternal wound infection, other types of infection could have occurred upon heart transplantation that crucially needs to be considered. Indeed, in-hospital post-operative infections are documented as the main cause of mortality and morbidity after cardiac transplantation [60]. Bacterial infections constitute the majority and also the most invasive type of postoperative infections [60].

An observational study on 677 adult patients who underwent heart transplantation from 1991 to 2015 revealed that the most common sources of infection included respiratory infections, urinary tract infections, bacteremia, abdominal focus, and surgical site infections which were related to invasive procedures [61]. Furthermore, Enterobacteriaceae and gram-positive cocci constitute the most frequent germs detected [61]. On the contrary, Shultes *et al*. reported that gram-negative bacteria were more common pathogens causing early post-operative infections. Moreover, they considered a history of drive-line infection, higher incidence of mechanical circulatory support, continuous renal replacement therapy, longer durations of ventilation and lengths of hospitalization, and delayed chest closure as common characteristics of cardiac transplantation recipients with early postoperative infection [62]. Importantly, another recent study revealed that multidrug-resistant (extended-spectrum beta-lactamase *Escherichia coli* and *Klebsiella pneumoniae*) and extensively drug-resistant (*Pseudomonas aeruginosa* and carbapenem-resistant *Klebsiella pneumoniae*) bacterial infections were seen in heart transplant recipients that caused bloodstream infection followed by pulmonary infection resulted in higher mortality rates [63]. In pediatric heart transplantation recipients, a study on 4458 patients from 1993 to 2014 revealed that early bacterial infection is the higher type of infection, with bloodstream infection as the most common infection site, and coagulase-negative staphylococci, Enterobacter sp. and Pseudomonas sp. as the most common pathogens [64]. Moreover, younger ages, as well as history in the use of ventilator and ECMO were acompanied by higher risks of infection causing high mortality rates [64]. In addition, a case report showed that Enterobacter cancerogenous could be identified in heart transplant recipients leading to early pneumopericardium [65]. Severe gastroparesis is a rare condition that could be diagnosed in recipients of orthotopic heart transplantation leading to significant morbidity, including vomiting, aspiration, and pneumonia [66]. Even more, viral infections such as severe acute respiratory syndrome coronavirus 2 (SARS-CoV-2) infection could cause acute biventricular heart failure symptoms following a post-myocarditis state in a heart transplantation recipient [67]. Fortunately, regarding viral infections, a large contemporary cohort study on heart transplant recipients demonstrated that cytomegalovirus infection was not associated with cardiac allograft rejection [68]. However, a retrospective cohort study from 2000 to 2009 on 966 patients who underwent heart transplantation depicted that renal replacement therapy and allograft rejection were related to invasive fungal and mold infections [69].

The broad-spectrum antibiotics and surgical management resulted in appropriate effects on in-hospital postoperative infection in patients who underwent heart transplantation [61]. Similarly, combined treatment, surgical and antimicrobial approaches, are required for the eradication of fungal endocarditis caused by *Candida parapsilosis* in patients who underwent cardiac surgery [70]. In addition, recent advances in preventive strategies and immunosuppression, such as pretransplant infectious diseases screening and antimicrobial prophylaxis, could be followed by a remarkable reduction in bacterial, viral, fungal, and Nocardia post-transplantation infectious episodes [62][69][71]. In spite of these effective treatment methods regarding multidrug-resistant and extensively drug-resistant bacterial infections that pivotally threaten one-year survival, the crucial need for further studies appeared to be extremely necessary [63].

*Low Cardiac Output Syndrome and Early Graft Dysfunction*

Graft dysfunction is defined as a failure of the transplanted heart to meet the requirements of the recipient’s circulatory in the immediate post-transplant period [72][73].

Primary graft dysfunction is considered a life-threatening condition and a major cause of early mortality in cardiac transplantation recipients. Indeed, primary graft dysfunction is documented as the leading cause of death after 30 days of transplantation, as it is the causative agent of 39% of early deaths. The severe dysfunction of the heart allograft without any clear anatomic or immunologic cause is the other definition of this condition, which is characterized by low cardiac output syndrome requiring mechanical or high-dose inotropic support [74].

Left ventricular diastolic diameter smaller than 36 mm, left ventricular ejection fraction less than 55%, and high inotropic requirement is considered as main risk factors for low cardiac output syndrome [75]. Moreover, donor age is a significant anticipator of low cardiac output syndrome that gradually caught up with those of early preserved output patients [76].

Taking together, suboptimal donor, poor donor management, old age, poor organ preservation, prolonged ischemia time, co-morbid illnesses, high baseline pulmonary artery pressure in the recipient, and long cardiopulmonary bypass time are documented as risk factors for low cardiac output syndrome after thoracic organ transplantation [77][78][79][80].

It is believed that low cardiac output after heart transplantation could be attributed to inadequate vasodilation, preload, and/or reduced inotropy [81][82]. Pollock *et al*. reported a case in which inadequate preload was due to an inferior vena cava thrombus [83]. Lim *et al*. revealed that early low cardiac power output index is extremely associated with severe primary graft dysfunction in which its serial assessment could dramatically increase the diagnostic probability of severe primary graft dysfunction [84].

Furthermore, low cardiac output syndrome may be accompanied by septic shock in patients who underwent cardiac transplantation, which could be tackled by early diagnosis and extensive antimicrobial therapy [85]. The veno-arterial extracorporeal membrane oxygenation is assumed as an effective approach to tackling primary graft dysfunction; however, mortality rates remained high [86][87].

Cautious donor selection by consideration of donor age, its left ventricular size, and ejection fraction, as well as inotropic, could be used to anticipate the possibility of low cardiac output syndrome and subsequent primary graft dysfunction after heart transplantation [75][76]. In the case of inferior vena cava thrombus leading to inadequate preload and subsequent low cardiac output syndrome, perioperative ultrasound could be used as a diagnostic approach [83].

*Pericardial Effusion and Constrictiv Pericarditis*

Pericardial effusion is described as an early complication following orthotopic cardiac transplantation that requires surgical interventions, which in turn prolongs hospitalization as well as increases the early mortality rate [88][89].

Moderate to severe pericardial effusion has been reported in more than 20% of patients during the first months; however, the progress of this complication later than one year after heart transplantation is rare [90].

Previous studies have suggested hemodynamically irrelevant pericardial effusion, which could be observed during common echocardiography, is associated with increased mortality in patients as a valuable predictor of adverse outcomes in patients with heart failure [91].

Interestingly, by assessment of 152 patients who underwent heart transplantation from 1989 to 2012, Stämpfli *et al*. revealed that pericardial effusion irrelevant to surgery in heart transplantation recipients could predict adverse outcomes as this early complication was associated with a 2.5-fold increased risk of recurrent hospitalization and mortality [92].

Furthermore, in a patient with dilated cardiomyopathy, a prevalent condition that commonly is followed by heart transplantation, a large pericardial effusion was revealed by a transthoracic echocardiogram, an atypical presentation which was accompanied by accumulation of a gross volume of fluid with no major cardiac compression or hypotension [93].

Importantly, a retrospective analysis of 25 patients who underwent heart transplantation by Lower-Shumway technique and administrated a standard triple immunosuppressive regimen including tacrolimus, mycophenolate mofetil, and prednisolone demonstrated that postoperative pericardial effusion following heart transplantation could be predicted by EuroSCORE as EuroSCORE more than 16% was a single predicting variable for postoperative pericardial effusion [94].

In addition, constrictive pericarditis is characterized as a disease with progressive cardiac fibrosis, inflammation, and thickening of the pericardium [95][96] that could follow any cardiac surgical procedure such as valvular replacement surgery, coronary bypass, corrective surgery for congenital heart disease, however, rarely following cardiac transplantation surgery [97][98].

Statistical analyzes state that the incidence rate of constrictive pericarditis varies from 1.5% to 4% [99]. Recently, Tchana-Sato *et al*. reported a patient who underwent heart transplantation followed by pericardial effusion due to idiopathic dilated cardiomyopathy leading to constrictive pericarditis, suggesting that postoperative pericardial effusion is a risk factor for further constrictive pericarditis [99]. 

Moreover, the authors stated that differentiation of pericardial effusion with other similar conditions in heart transplantation recipients is a challenge and surgical pericardiectomy is the main management approach for constrictive pericarditis [99].

Concordantly, Bansal *et al*. demonstrated 5 cases with postoperative early pericardial effusion of non-infectious etiology leading to constrictive pericarditis and, subsequently, heart failure unresponsive to standard medical strategies. The findings determined that the ideal use of Doppler echocardiography and a high index of clinical suspicion are required for early diagnosis of constrictive pericarditis following heart transplantation and surgical pericardiectomy is the effective therapeutic strategy [100].

*Early Rejection*

What could be concluded from the previous sub-headings, the current clinical knowledge has tackled postoperative complications in heart transplant recipients through advances in immunosuppressive treatments, novel surgical techniques, donor heart procurement, donor-recipient compatibility, and post-transplantation care. For instance, by the analysis of donor heart selection outcomes in more than 2000 recipients, the researchers concluded that less restrictive but highly proportionate policies for accepting donor’s hearts, particularly regarding high catecholamine levels, rejection for positive virology or non-quality reasons, low ejection fraction, and longer ischemic time could lead to both expansion of donor pool and maintaining desirable results [101].

In addition, Katz *et al*. assessed 216 patients who underwent heart transplantation and revealed that the risk of early rejections following heart transplantation is remarkably dropped over the past twenty years [102]. Improvements in immunosuppressive therapy, for example, in induction therapy by novel interleukin-2 antagonists, resulted in suitable survival with an almost 25% reduction in absolute and relative risks, as well as dropping rejection rates [103].

Although all the mentioned solutions have caused a gradual reduction in both acute allograft rejection and a more appropriate survival rate following cardiac transplantation over time, 10- 30% of cardiac transplant recipients undergo early allograft rejection within the first year post-transplantation [104].

Acute cellular rejection, one of the main remained postoperative complications after heart transplantation, is defined by an inflammatory infiltrate possibly associated with damage to the heart [105].

This complication is assigned grades of 0-4, based on the cause of infiltrating or the damage to myocardial and vascular components, by the international society for heart and lung transplantation [106].

An autopsy study on 39 patients following heart transplantation demonstrated that early cellular rejection is the cause of death in 8% of studied patients [107].

A recent study in 2021 reported that coronary microcirculatory dysfunction, revealed by the index of microcirculatory resistance (IMR) early after heart transplantation, is highly associated with the risk of acute cellular rejection [108].

In addition, another recent study showed that the weight gain in recipients after heart transplantation could increase the risk for allograft vasculopathy and rejection, suggesting the impact of obesity on outcomes of heart transplantation [109].

The number of previous rejections, elapsed time since the previous rejection, and race is considered risk factors for recurrent rejection requiring retransplantation [110].

Nonetheless, over the past decade, several studies suggested novel diagnostic techniques and biomarkers to assess and follow heart transplantation recipients and predict the risk of early rejection [111][112].

## Conclusion

To put all findings in a nutshell, continuous studies indicate the advances made in the recent two decades, such as improvements in immunosuppressive pharmaceuticals, determining appropriate policies for donor acceptance, pre- and post-operative treatment/care, selection of the most compatible donor with the recipient, the presence of novel diagnostic and surgical techniques, and so forth, have remarkably declined the early postoperative complications after heart transplantation. Nevertheless, the rate of mortality early after transplantation remains high due to remained complications such as arrhythmia, atrial fibrillation, atrial flutter, deep sternal wound infection, low cardiac output syndrome, early graft dysfunction, pericardial effusion, constrictive pericarditis, and early rejection which require further investigations.

## Conflict of Interest

The authors have no conflict of interest to declare.
